# Subchronic toxicity of magnesium oxide nanoparticles to *Bombyx mori* silkworm†

**DOI:** 10.1039/d2ra01161a

**Published:** 2022-06-10

**Authors:** Lin Ma, Vivian Andoh, Zhongyuan Shen, Haiyan Liu, Long Li, Keping Chen

**Affiliations:** College of Biotechnology, Jiangsu University of Science and Technology Zhenjiang Jiangsu 212001 P. R. China ma_lin_1988@126.com; Institute of Life Science, Jiangsu University Zhenjiang Jiangsu 212013 P. R. China kpchen@ujs.edu.cn; Tea and Food Science and Technology Institute, Jiangsu Vocational College of Agriculture and Forestry Jurong 212400 China

## Abstract

Despite many research efforts devoted to the study of the effects of magnesium oxide nanoparticles (MgO NPs) on cells or animals in recent years, data related to the potential long-term effects of this nanomaterial are still scarce. The aim of this study is to explore the subchronic effects of MgO NPs on *Bombyx mori* silkworm, a complete metamorphosis insect with four development stages (egg, larva, pupa, month). With this end in view, silkworm larvae were exposed to MgO NPs at different mass concentrations (1%, 2%, 3% and 4%) throughout their fifth instar larva. Their development, survival rate, cell morphology, gene expressions, and especially silk properties were compared with a control. The results demonstrate that MgO NPs have no significant negative impact on the growth or tissues. The cocooning rate and silk quality also display normal results. However, a total of 806 genes are differentially expressed in the silk gland (a vital organ for producing silk). GO (Gene Ontology) results show that the expression of many genes related to transporter activity are significantly changed, revealing that active transport is the main mechanism for the penetration of MgO NPs, which also proves that MgO NPs are adsorbed by cells. KEGG (Kyoto Encyclopedia of Genes and Genomes) analysis demonstrates that the longevity regulating pathway-worm, peroxisome and MAPK signaling pathway are closely involved in the biological effects of MgO NPs. Overall, subchronic exposure to MgO NPs induced no apparent negative impact on silkworm growth or silks but changed the expressions of some genes.

## Abstract 2

The subchronic toxicity of MgO NPs was studied by silkworm model, from the levels of animal entirety, tissues, and genes.

## Graphical abstract

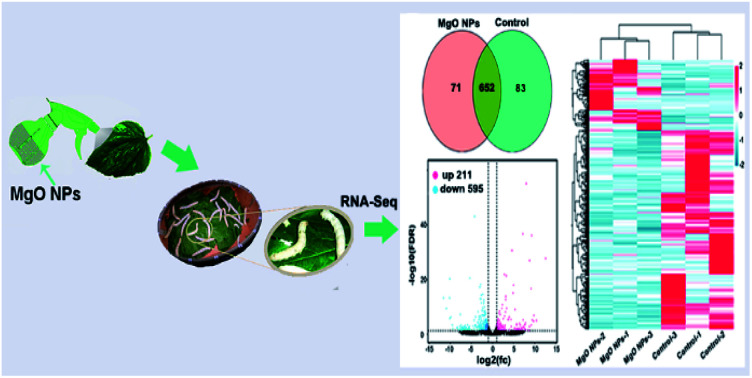

## 1 Introduction

Magnesium oxide nanoparticles (MgO NPs) are in widespread use in various fields including (but not limited to) corrosion inhibitors, refractory fiber boards, antibacterial agents, anti-cancer therapy and so on, due to their unique properties, easy synthesis and chemical stability.^1–3^ For example, due to their excellent anti-UV properties, MgO NPs are widely applied in the cosmetics and clothing fields. This material is also favored by the ceramics industry because it reveals that porcelains containing MgO NPs have the ability of water purification, anti-bacterial properties and also release beneficial ions.^4^ MgO NPs are also demonstrated to be good additives in heavy fuel-oil due to their high oil dispersion ability.^5^ As more MgO NPs are produced and commercialized, there are several ways for them to enter people's lives. They can be released into the environment by industrial processes, wastewater sludge, and waste dumps, as well as natural occurrences like dust storms, wildfires, and volcanoes.^6^ MgO NPs can also be introduced in the environment during the stages in the life-cycle of products containing MgO NPs such as transportation, production, washing *etc.*^7,8^ It was reported that NPs could penetrate biofilms and adsorb on the surface of biomolecules in cells,^9,10^ thus it is essential to evaluate the environmental health and safety of MgO NPs with their increasing application.

Some groups have investigated the *in vitro* cytotoxic effects of MgO NPs,^11–15^ for example, Ge and coworkers^11^ reported the cytotoxic effects of MgO NPs on human umbilical vein endothelial cells, Krishnamoorthy *et al.*^12^ investigated the mechanism of the toxicity of MgO NPs on cancerous cells, Mahmoud *et al.*^13^ studied the toxic effects of MgO NPs on lung (A549), kidney (NRK-52E), liver (HepG2) and colon (Caco-2) cell lines, Sun *et al.*^14^ studied the *in vitro* cytotoxicity of MgO NPs in human cardiac microvascular endothelial cells, Jebali *et al.*^15^ compared the antileishmanial activity and toxicity between lectin-coated and uncoated MgO NPs, by observing the macrophages of BALB/c mice and promastigotes of *Leishmania major*. Compared with in the *vitro* study, *in vivo* evaluation of NPs is more important because the interactions between NPs and animal systems are rather complicated, which may cause novel immune response or metabolism patterns *et al.* and thus provide more useful information on their likely health hazards to human beings.^1^ Several groups have carried out *in vivo* studies to assess the biological effects of MgO NPs.^1,16–20^ Mangalampalli *et al.*^1^ carried out an acute oral toxicity study of MgO NPs in female albino Wistar rats and pointed out that acute exposure to large dosage of MgO NPs lead to obvious biochemical alterations and DNA damage. Kiranmai and coworkers^16^ reported that through acute intratracheal instillation into rat with MgO NPs, a reduction of the total antioxidant capacity in serum was found. Ghobadian *et al.*^17^ studied the toxic effects of MgO NPs by incubating zebrafish embryo in MgO NPs solution and found that MgO NPs greatly affected the survival and hatching rates of zebrafish embryos and caused a lot of malformations. Mangalampalli and coworkers^18^ evaluated the toxicity of MgO NPs in Wistar rats by a 28 day oral administration and demonstrated that MgO NPs changed the activities of the biochemical enzymes. Gelli *et al.*^19^ assessed the *in vivo* toxicity of MgO NPs in rats *via* acute intratracheal instillation and found that MgO NPs resulted in pulmonary toxicity in rats. Kovrižnych *et al.*^20^ evaluated the acute toxicity of 31 various NPs including MgO NPs on fish mature individuals *Danio rerio* and revealed that MgO NPs could cause cumulative mortality. Although *in vivo* exposures were already investigated, chronic or subchronic toxicity study on organisms is still scarce compared with acute evaluation. Our study aims at evaluating the effects of MgO NPs in a model invertebrate organism, *Bombyx mori* silkworm, by an 8 day exposure throughout the whole fifth instar of silkworm larva.

Silkworm (*Bombyx mori*) is a representation of lepidopteran insect and a good model organism. It is a complete metamorphosis insect with four development stages (egg, larva, pupa, month), and the larva stage (in total about 30 days) is divided into 5 instars based on the size. As a kind of invertebrates, silkworm will not lead to ethical issues compared with mammalian models like rats, mice and rabbits, in addition, it is also reported that silkworm has similar median lethal doses and common pharmacokinetics with mammals.^21–23^ Compared to the typical non-mammalian models like *Drosophila*, *Salamandra Laurenti*, and zebrafish, the silkworm cannot survive outside and thus will not cause problems of biosafety, besides, the silkworm has a modest size which is easy for dissection.^23,24^ Moreover, the silkworm also has many other advantages, for example, short generation time, great genetic resources accompanied by various morphological mutation, and high sensitivity to toxins.^23,25^ Due to its outstanding virtues, the silkworm is flavored by researchers from many fields such as classical genetics, human disease investigation, medicinal studies, and environmental monitoring.^26–31^ For instance, researchers have discovered that some genes associated with human genetic diseases possess high sequence similarities with *Bombyx mori* silkworm genes, based on which models of human genetic diseases (including but not limited to Phenylketonuria, Hermansky-Pudlak syndrome, Parkinson's disease) can be created.^32–35^ Silkworm model is also hot in the field of nanomaterials toxicity evaluation in recent years, various NPs were evaluated including metal NPs, carbon NPs and semiconductor quantum dots and so on.^23,32,36–42^

In this paper, the fifth-instar silkworm larva was chosen as a model to investigate the effects of MgO NPs, we chose larva at this stage because among all the stages, the fifth-instar silkworm larva has the biggest appetite and fastest eating speed. Through an 8 day oral administration to silkworm larva, the subchronic toxicity of MgO NPs was evaluated from the levels of animal entirety, tissues and genes. The effects of MgO NPs on the animal entirety were evaluated by studying the survival rate, growth (appearance, length and weight, cocoon appearance) and silk properties (morphology, diameter, secondary and crystalline structures, mechanical properties). The impacts of MgO NPs on tissues were studied by histopathological examination. RNA sequencing method (RNA-seq) was used to study the influence of MgO NPs on the gene expressions and reveal the possible mechanism underlying their effects.

## 2 Experimental section

### 2.1. Materials and reagents

“Jingsong × haoyue” *Bombyx mori* silkworm eggs were got from Shandong guangtong silkworm egg group company. Magnesium oxide nanoparticles (MgO NPs) powder with an average diameter of about 20 nm were from Nanjing XFNANO Materials TECH Co., Ltd. The characterizations of the purchased MgO NPs were provided in ESI.† Deionized water was provided by an Elix5+Milli-Q water purification machine (Millipore, USA).

### 2.2. Characterizations

The raw data from inductively coupled plasma mass spectrometer (ICPMS), scanning electron microscope (SEM) and Fourier-transform infrared spectra (FTIR) were supplied by Shanghai Yuyi Analysis and Detecting Center: ICPMS (Agilent 7700, America) was used for detecting the content of Mg element in silkworm organs and silks (three replicates were acquired for each sample), a MIRA3 SEM (Tescan, Czech Republic) was used to study the surface and size of silks, a Thermo Fisher NICOLET 6700 FTIR spectrometer, equipped with a diamond ATR accessory, was used to collect the FTIR data. Wide angle X-ray scattering raw data were provided by Physical Science and Technology College from Xiamen University. The mechanical parameters of the degummed silks were collected by textile and clothing engineering college from Soochow University, *via* an Instron 3365 universal testing machine (Instron, America). Jiangbin hospital in Zhenjiang city helped to make silkworm organs into tissue sections. Briefly, the tissues were dehydrated by a serial of alcohol solutions and embedded in paraffin, which were cut into 5 μm-thick sections by a microtome. The sections were then dewaxed with xylene and rehydrated *via* decreasing concentrations of ethanol, which were then stained with hematoxylin and eosin (H&E). A German Leica EZ4HD microscope was used for histophysiological analysis. Silk glands were taken and saved under −80 °C after exposed to MgO NPs for 120 hours for RNA-Seq analysis, supported by Genedenovo Biotechnology company in Guangzhou city of China.

For the comparison of the average weight (AW) and length (AL) of larvae between control and each MgO NPs-group, pooled variances *T*-test (Student's *t*-test) and separate variance *T*-test were performed based on the results of *F*-test, using the data analysis package in Microsoft Excel (Office 365). Origin 8.5 and Adobe Photoshop CS2 were used for the processing of nearly all the graphs and pictures.

### 2.3. The intake of MgO NPs by silkworm

Uniform dispersed MgO NPs suspension solutions at different mass concentrations (1.0%, 2.0%, 3.0% and 4.0%) were prepared by mixing MgO NPs (1.0 g, 2.0 g, 3.0 g and 4.0 g) with water (0.1 L), respectively, followed by 20 min ultrasonication.

4 groups of silkworms (20 in each) were fed by MgO NPs from the 1st day of 5th instar until cocooning, by spraying NPs solution onto mulberry leaves. The concentration of the NPs is the same within groups while different among groups. 20 silkworms fed with normal mulberry leaves were set as control. The whole process lasted for 8 days. According to rough statistic, each larva takes about 2 g mulberry leaves each time, the mass ratio of mulberry leave to MgO NPs is approximately 10 000 : 27, 10 000 : 54, 10 000 : 81 and 10 000 : 108 as the mass concentration of NPs increases from 1% to 4%.

More details about tensile test, silkworm rearing, and silk reeling can be seen in ESI (Experimental section†).

## 3 Results and discussion

### 3.1. The survival rate and distribution of MgO NPs in silkworm

In this assay, 5 groups of silkworm larva with 20 in each were fed by MgO NPs with various mass concentrations, group 1 with 1%, group 2 with 2%, group 3 with 3%, group 4 with 4%, control with 0%. In the following statement, G1–G4 is used for short instead of group 1–4. The survival rate was recorded every 24 h during the feeding of MgO NPs. No death was observed during the whole process, showing that within a certain concentration, the exposure of silkworm larvae to MgO NPs is of low risk.

ICP-MS experiment was performed to study the distribution of Mg element in silkworm silk, midgut, fat body, and silk gland after the intake of MgO NPs for 120 h (G4 and control were used as examples). Three replicates were tested for each sample. The results in Table 1 show that the contents of Mg in silk from control and G4 groups are comparable, indicating that Mg is not accumulated in silk. It is also found that the distribution of Mg in silkworm organs exhibits a significant change, compared with control, the Mg contents in G4-midgut and G4-silk gland are increased obviously, while that in G4-fat body is decreased substantially. The results show that the continuous intake of MgO NPs has a notable influence on the distribution of Mg in the silkworm body, but no predominant accumulation of Mg is observed, indicating that MgO NPs can be easily cleared and may affect the metabolism process of the silkworm.

**Table 1:** The contents of Mg element in different organs and silk of silkworms (mg kg^−1^)

Sample	Midgut	Fat body	Silk gland	Silk
G4	1500.00 ± 100.00	466.67 ± 5.77	886.67 ± 15.28	310.00 ± 10.00
Control	853.33 ± 15.28	1266.67 ± 57.74	580.00 ± 10.00	316.67 ± 5.77

### 3.2. Effect of MgO NPs on the growth and silks of silkworms

#### 3.2.1. Effect of MgO NPs on the growth, cocoon, and silk morphology of silkworms

**Fig. 1:**
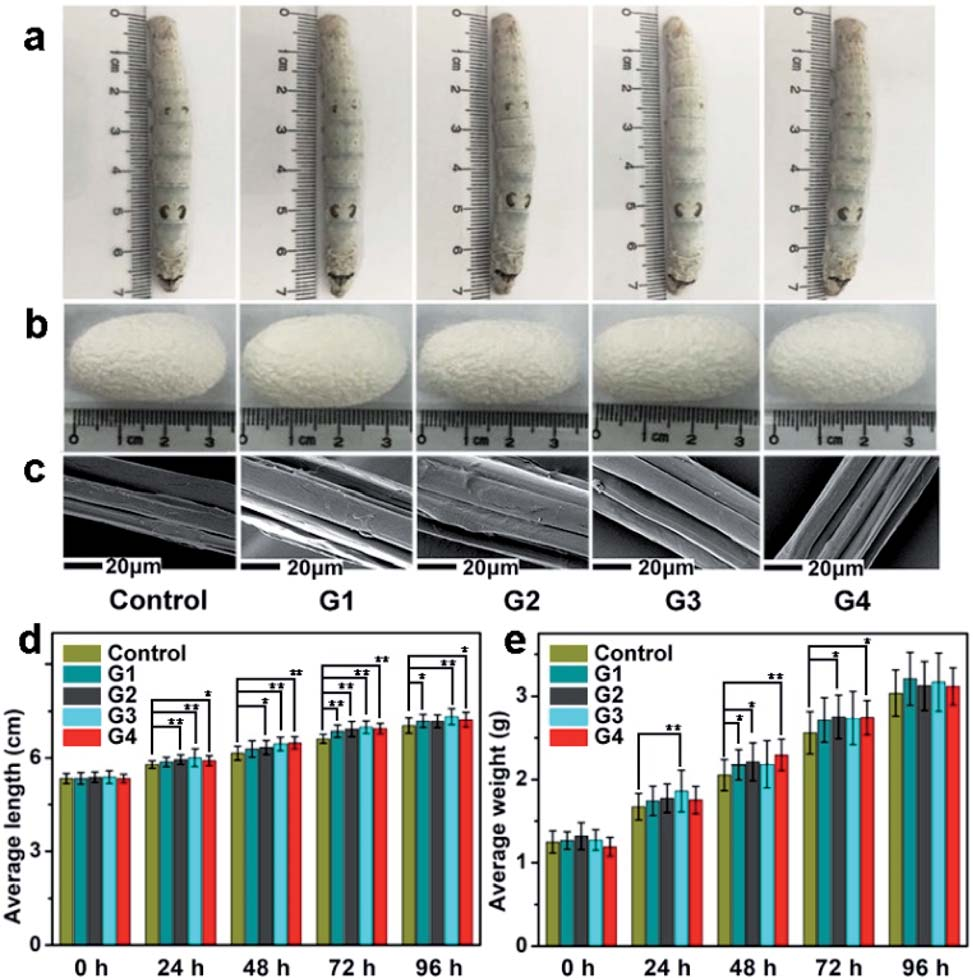
The influence of MgO NPs on the growth, cocoons as well as the morphology of silks of silkworms. (a) The influence of MgO NPs on the exterior of silkworm larvae after the feeding of MgO NPs for 96 h (b, c) The influence of MgO NPs on the cocoons (b) and silks (c). (d, e) The influence of MgO NPs on the AL (d) and AW (e) of silkworm larvae after the feeding of MgO NPs for different times. Error bars refer to the standard deviation of silkworm length (d) and weight (e). *P < 0.05 and **P < 0.01 indicate significant differences.

We observed the body appearance (Fig. 1a), weight (Fig. 1d) and length (Fig. 1e) at 24 h intervals to evaluate the effect of MgO NPs on the growth of silkworms. The average values for the larva weight and length exhibited in Fig. 1d and e were calculated from twenty specimens for each group. Student's *t*-test (*t*-test) was used to assess the difference of the average length and weight (AW and AL) between control and each MgO NPs-group. Fig. 1a shows that larvae from both control and MgO NPs groups exhibit similar exteriors. From Fig. 1d and e, it is observed that the values of AL and AW from all the groups are very close, although the ones from MgO NPs-groups are slightly higher. Statistically significant difference is found between control and some MgO NPs-groups. However, statistical significance does not equal practical importance.^43,44^ The photos of cocoons form different groups (Fig. 1b) show that MgO NPs cause no significant influence on the cocoon appearance, the AW of the cocoons from the 5 groups (Fig. S3a†) were also found to be comparable, no obvious change was observed.

The morphology and average diameter (AD) of the silks from the cocoons were also investigated by SEM (the silks studied were reeled silks, meaning that the sericin coated out of the layer of silks were removed), the results are shown in Fig. 1c and S3b.†Fig. 1c indicates that MgO NPs cause no obvious change to the morphology of the silks, it is also found that the AD of silks from every group is similar (Fig. S3b†). In general, MgO NPs have no obvious negative effects on the growth, cocoons and silk morphology of the silkworm.

#### 3.2.2. The effect of MgO NPs on the structure and mechanical properties of silks

Silk fibroin is the main product of silkworm, the quality of which can strongly reflect the health of silkworm larvae. When silkworm larvae adsorb poisonous substances, its cocooning rate and silk quality will decrease a lot. Thus, the structure and mechanical properties of silks are investigated in the assay.

##### 3.2.2.1 Crystalline and secondary structure

**Fig. 2:**
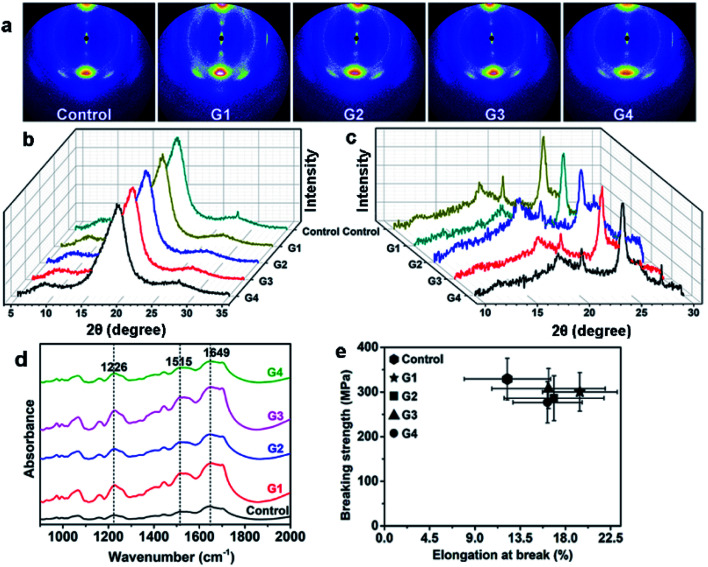
(a–c) WAXS patterns of different silks: (a) 2D-WAXS patterns, (b, c) 1D-WAXS profiles along the equatorial (b) and meridian (c) direction of the 2D patterns. (d) FTIR spectra of different silks. (e) The elongation at break-breaking strength behavior of silks from different groups, the error bars refer to the standard deviation of breaking strength (ordinate) and elongation at break (abscissa).

The crystalline structure of silks was studied by wide angle X-ray scattering (WAXS) method, the results are shown in Fig. 2a–c. It is found that both 2D and 1D results of all the silks show no distinct difference, suggesting that crystalline structure is not damaged by MgO NPs. The secondary structure of the silks was also studied by FTIR analysis, and the results are shown in Fig. 2d. It shows that the FTIR spectra of all the silks do not show any significant difference, indicating the fine preservation of the internal structures of silks, which is in accordance with the WAXS results. Three predominant peaks located at 1226 cm^−1^ (amide III, assigned to random coil or helix or both), 1515 cm^−1^ (amide II, assigned to β-sheet) and 1649 cm^−1^ (amide I, assigned to random coil or helix or both) are found, meaning the co-existence of β-sheet and helix/random coil conformations.^23,32,36^

##### 3.2.2.2 The mechanical properties of silks

The influence of MgO NPs on the mechanical properties of silks was also studied, by the analysis of the elongation at break (EB), breaking strength (BS) and toughness modulus (TM) of different silks. Twenty specimens were measured for each group, *i.e.* the statistical data for every groups was the results from twenty repeats. The EB–BS behaviour and stress–strain curves of various silks are demonstrated in Fig. 2e and S4,† respectively. The values of average EB, BS and TM (the area beneath the stress–strain curve) of different silk samples are shown in Table S1.† It is found that BS of silks from each MgO NPs group is similar but is a little lower than that from control group. On the contrary, EB from each MgO NPs-group is much higher than the one from control, which may be attributed to the increase of the helix/random coil contents in silks.^23,36^ More experiments need to be performed to elucidate the mechanisms. Overall, the intake of MgO NPs does not cause serve damage to the silk's mechanical properties.

### 3.3. Histophysiological examination

**Fig. 3:**
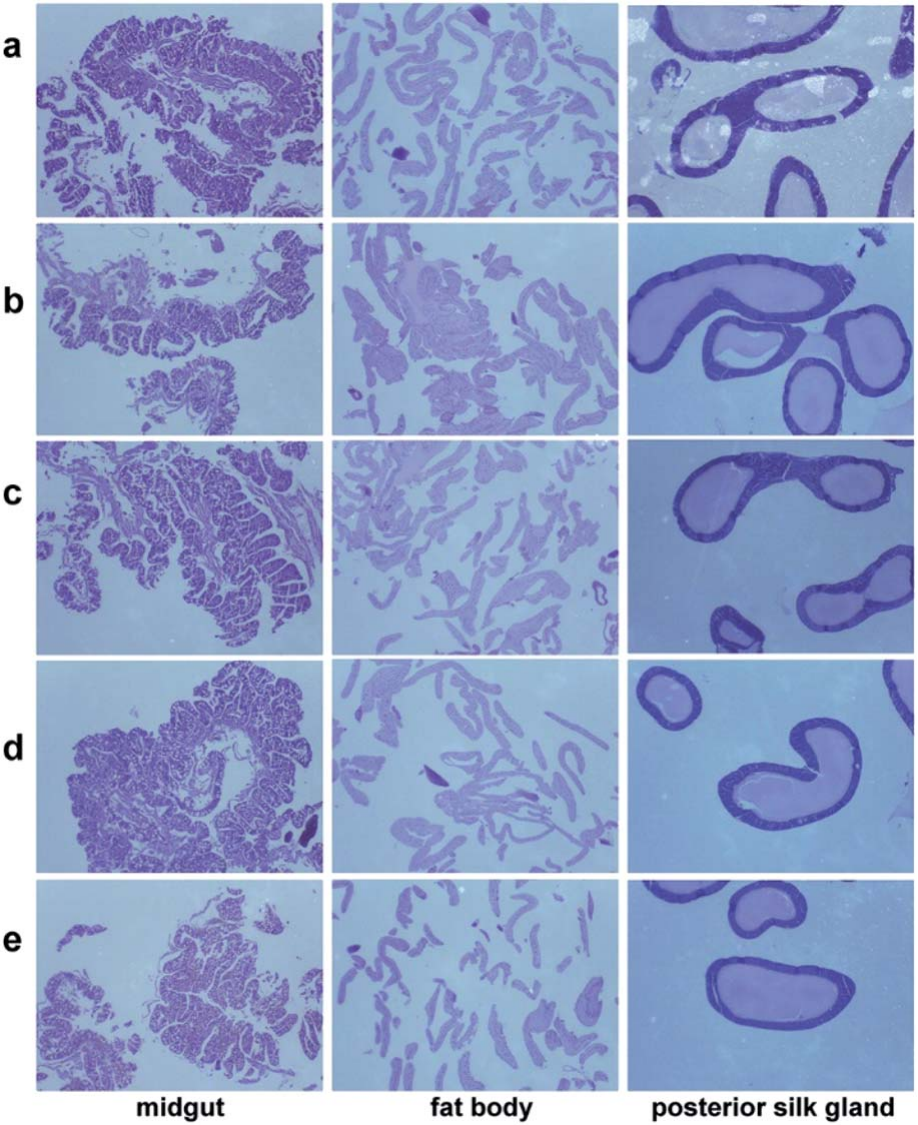
Histophysiological images of the midgut, fat body, and posterior silk gland from different groups after feeding silkworms with MgO NPs for 96 h: (a) control, (b) G1, (c) G2, (d) G3, (e) G4. The magnification is 35 times.

Histophysiological study of silkworm tissues (midgut, fat body and posterior silk gland), was performed by observing their paraffin sections stained with hematoxylin and eosin (H&E), the results are shown in Fig. 3. It displays that MgO NPs do not influence the pathological microstructures of the silkworm. Midguts from all groups exhibit normal structure with distinct basal laminae. Fat body cells from every group are finely ordered with even distance between each other, indicating that no destroy is caused. Posterior silk glands from both groups show no pathological difference, which have thin walls and full lumen.

### 3.4. Global gene expression changes

**Fig. 4:**
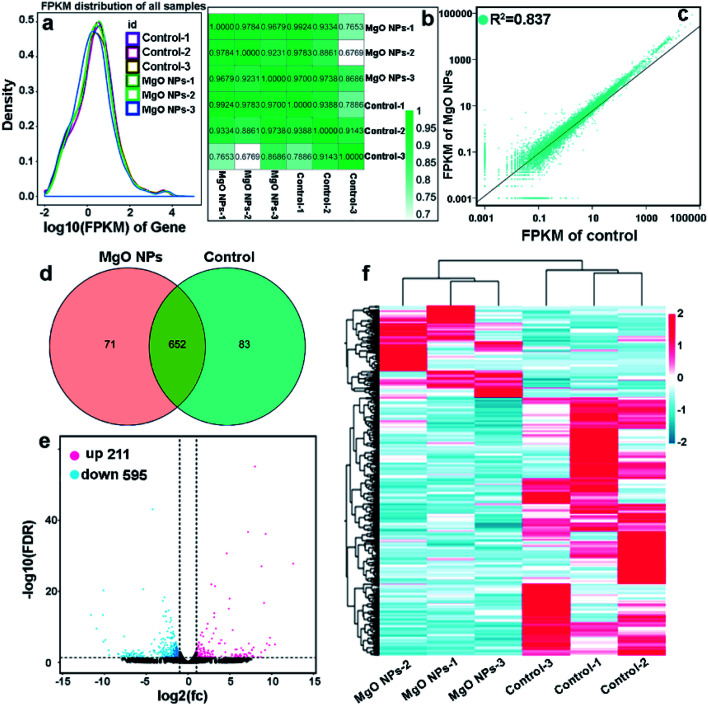
(a) Comparison of gene expression levels of control and MgO NPs groups. (b) Heat map diagram of Pearson correlation coefficient between control and MgO NPs groups. (c) Scatter diagram of Pearson correlation coefficient between control and MgO NPs groups. (d) Venn-diagram showing overlapping sets of DEGs obtained for control and MgO NPs. (e) Volcano plot of DEGs between control and MgO NPs groups. (f) Hierarchical clustering of DEGs in control and MgO NPs groups. Each row refers to a single gene and each column refers to one sample. Red color means up-regulated, blue color means down-regulated.

RNA-seq based transcriptional analysis of silk gland from control and MgO groups (took G1 as an example) was performed to know the impact of MgO NPs on the gene expression of the silkworm. In order to identify the deferentially expressed genes (DEGs), the expression of all the genes were analyzed by FPKM (Fragments Per Kilobase Million), the expected number of fragments per kilobase of transcript sequence per million base pairs sequenced.^45^ The gene expression levels were analyzed according to universal reads, and the differential gene expression was calculated through the DESeq2 software. The result in Fig. 4a shows that most genes in both control and MgO NPs groups possess similar distribution of RNA-seq read counts, indicating that the treated-samples were not polluted by other species. Heat map diagram and linear regression analysis were used to assess the association between MgO NPs and control samples (Fig. 4b and c), through Pearson's correlation coefficient (*R*). The *R* value between control and MgO NPs samples is 0.915, *R*^2^ value (0.837) ˃ 0.8 presents a good-level of correlation. Venn diagrams of DEGs between control and MgO NPs groups are shown in Fig. 4d, a total of 806 DEGs (*P* ˂ 0.05, FDR ˂ 0.05, |log2(fold change)| > 1) are found, among which 652 transcripts are commonly expressed in the two groups. Out of these genes, 83 and 71 DEGs were identified in MgO NPs and control groups, respectively. Compared with control, there are 211 up-regulated and 595 down-regulated genes in MgO NPs group (Fig. 4e). DEG expression levels in the control and MgO NPs groups were plotted in a heat-map in Fig. 4f.

#### 3.4.1. Expression changes of genes about silk

Genes related to silk with statistically significant differential expression (*P* ˂ 0.05) were first investigated. From Table 2, it is obtained that the genes corresponding to fibroin (fibroin heavy chain precursor, fibroin light chain precursor, and P25) are up-regulated to some extent, the sericin 1-like gene related to sericin is also up-regulated while sericin 2 gene is down-regulated. Generally, MgO NPs cause no obvious negative effect on the genes related to the production of silk.

**Table 2:** The expression changes of genes related to silk

ID	Fold change (G1/control)	*P* value	FDR	Description
MSTRG.18835	2.05	1.41 × 10^−5^	0.000723	Fibroin heavy chain precursor [*Bombyx mori*]
ncbi_693030	1.45	0.0234	0.126	Fibroin heavy chain precursor
ncbi_693047	1.64	0.00271	0.0315	Fibroin light chain precursor
ncbi_100146105	1.65	0.00242	0.0296	P25
ncbi_105842827	1.45	0.0236	0.127	Sericin 1-like
ncbi_101744589	1.43	0.0280	0.142	Sericin 1-like
ncbi_100379325	0.602	0.00202	0.0259	Sericin 2

#### 3.4.2. Expression changes of genes about metabolism, immune and xenobiotics biodegradation

The significant expression changes of genes related to metabolism, immune and xenobiotics biodegradation were also studied, the results can be seen in Tables S2–S4.† It is observed that 95 genes related to metabolism (27 up-regulated, 68 down-regulated), 23 genes related to immune (7 up-regulated, 16 down-regulated) and 18 genes related to xenobiotics biodegradation (5 up-regulated, 13 down-regulated) were significantly affected by MgO NPs, indicating that MgO NPs have significant influence on the functions of metabolism, immune and xenobiotics biodegradation of silkworm, among which the number of genes affected concerned with metabolism is the largest, indicating that MgO NPs may have potential danger to cause dysfunction.

It is speculated that the RNA-seq results from higher-dose groups (G2, G3 and G4) would show a more comparative and vivid assessment. The number of the DEGs may increase with more significant *P*-values, especially the ones related to metabolism, immune and xenobiotics biodegradation. The expression of some genes corresponding to silk production maybe down-regulated based on the results of the mechanical properties. However, further experiments need to be done to prove the conjecture in the future study.

#### 3.4.3. Gene ontology (GO) enrichment analysis

**Fig. 5:**
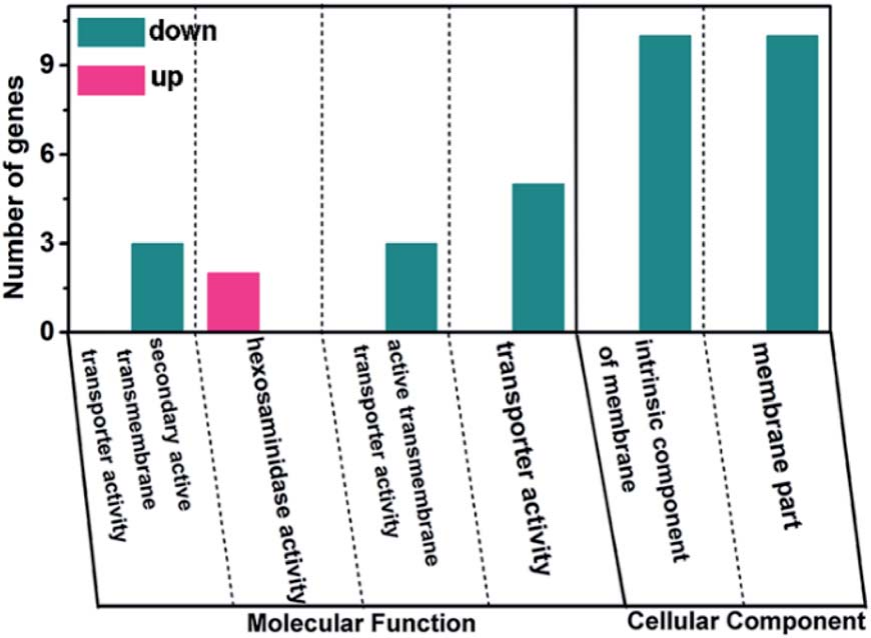
GO terms of molecular function and cellular component terms significantly affected by MgO NPs (*P* < 0.05).

GO is a system which can provide classifications and vocabularies for the annotation of genes.^46,47^ In order to characterize the functions of all the DEGs, GO enrichment analysis was performed. The results (Table S5†) were divided into three categories as biological process (BP), molecular function (MF) and cellular component (CC). The most significant terms (*P* ˂ 0.05) are shown in Fig. 5, it displays that the most highlighted terms in MF group are secondary active transmembrane transporter activity, hexosaminidase activity, active transmembrane transporter activity and transporter activity, while intrinsic component of membrane and membrane part are the most notable terms in CC group. It was reported that NPs can be internalised by cells *via* active transport mechanisms such as endocytosis *etc.*, which can also be internalised *via* passive diffusion if small enough (˂20 nm).^48^ The GO results show that the expression of many genes related to transporter activity are significantly changed, revealing that active transport is the main mechanism for the penetration of MgO NPs, which also proves that MgO NPs is adsorbed by cells.

#### 3.4.4. Kyoto encyclopedia of genes and genomes (KEGG) pathway analysis

**Fig. 6:**
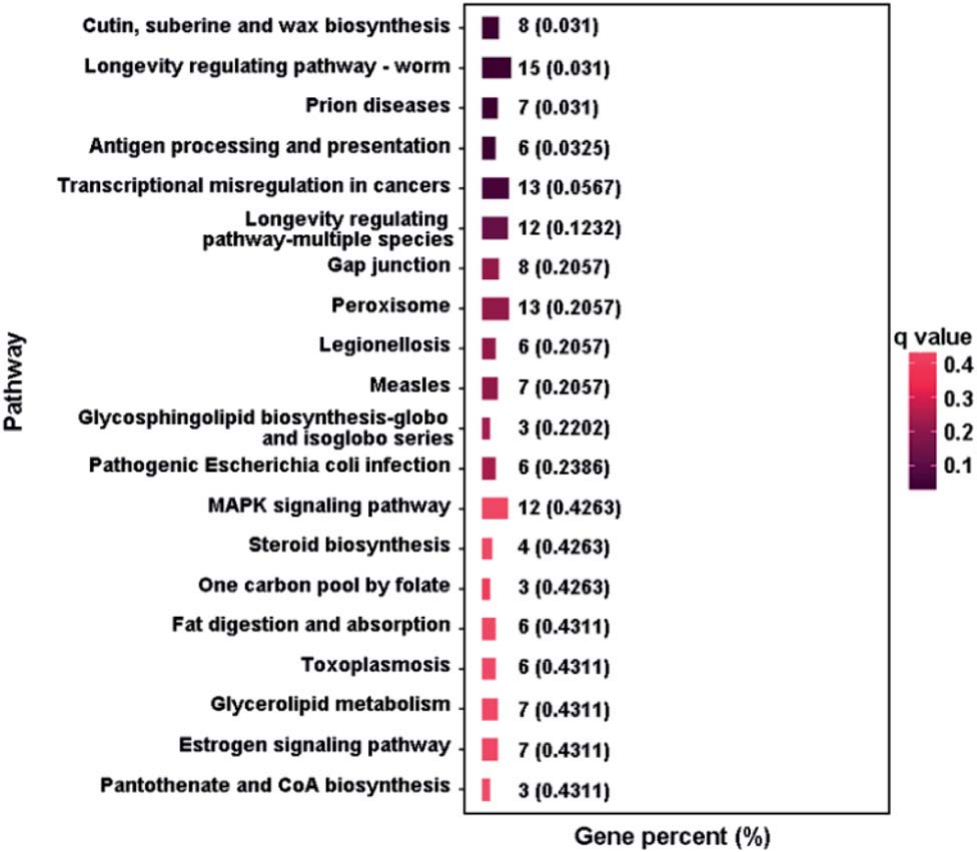
KEGG pathway analysis of differently expressed genes (TOP 20 of the most significant KEGG enrichment).

KEGG is a bioinformatics database that could be used for the analysis of gene functions, which provides useful classification for comprehending the complicated biological functions of genes.^47^ In order to elucidate the DEGs in different pathways, KEGG enrichment analysis was performed on the DEGs. The results (Table S6†) show that 199 DEGs are divided into 26 significant pathways (*P* ˂ 0.05), among which 138 genes are down-regulated and 61 genes are up-regulated. 10 pathways belong to metabolism, 6 are associated with organismal systems, 6 are related to human disease, 2 are relevant to cellular process, 1 is connected with environmental information and 1 is about genetic information processing. The TOP 20 KEGG pathways are shown in Fig. 6, it is observed that the largest number of genes of pathways include longevity regulating pathway-worm (15, 7.54%), transcriptional misregulation in cancers (13, 6.53%), peroxisome (13, 6.53%), longevity regulating pathway-multiple species (12, 6.03%) and MAPK signaling pathway (12, 6.03%), indicating their important roles to MgO NPs uptake. The pathways of longevity regulating pathway-worm and longevity regulating pathway-multiple species are related to aging, transcriptional misregulation in cancers is about human disease, peroxisome is connected with transport and catabolism, MAPK signaling pathway is relevant to signal transduction.

The peroxisome pathway plays a key role in redox signaling and lipid homeostasis and is also associated with antioxidant systems, which includes peroxisome biogenesis and peroxisomal proteins composed of 7 processes (fatty acid-oxidation, etherphospholipid biosynthesis, antioxidant system, sterol precursor biosynthesis, purine metabolism, amino acid metabolism and retinol metabolism),^47,49,50^ see Fig. S5.† In this pathway, the acyl-CoA oxidase (ACOX) and catalase (CAT) genes are down-regulated, the dehydrogenase/reductase family member 4 (DHRS4) gene is up-regulated, the alcohol-forming fatty acyl-CoA reductase (FAR) gene displays mixed regulation. The expression changes of ACOX, DHRS4 and FAR are related with fatty acid-oxidation, retinol metabolism and etherphospholipid biosynthesis, respectively, which may cause growth disorder to silkworm larvae. The down-regulation of the gene of CAT, the crucial antioxidant enzyme which could scavenge ROS and decompose H_2_O_2_,^47,51,52^ indicates that MgO NPs may have a negative impact on the antioxidant system of silkworm.

## 4 Conclusions

In this paper, the subchronic biological effects and possible mechanism of MgO NPs are studied by using silkworm as a model animal, which also provides data to enrich their toxicological database. It reveals that MgO NPs in a certain range do not influence the growth condition, silk properties and cell morphology of the silkworm. The RNA-seq data of silk gland exhibit that the expression of genes about producing silk fibroin is up-regulated, indicating that MgO NPs cause no negative influence on the production of silk, the expressions of genes about some other important functions (metabolism, immune and xenobiotics biodegradation) change significantly, showing that MgO NPs could lead to dysfunction as a potential danger. GO analysis shows that the interaction between MgO NPs and cells are based on transporter activity, KEGG analysis demonstrates that longevity regulating pathway-worm, peroxisome and MAPK signaling pathway are closely involved in the biological effects of MgO NPs.

## Author contributions

Lin Ma designed the experiment, provided financial support, and wrote the whole paper. Vivian Andoh conducted the experiments. Zhongyuan Shen provided experiment place and gave guidance about rearing silkworms. Haiyan Liu helped to check the grammar and spelling mistakes. Long Li provided financial support. Keping Chen provided technique support and writing guidance. Each author contributed to the general discussion.

## Conflicts of interest

There are no conflicts to declare.

## Supplementary Material

RA-012-D2RA01161A-s001
