# Supplementary material for: Subchronic toxicity of magnesium oxide nanoparticles to Bombyx mori silkworm
Source: RSC Adv. 2022 Jun 10;12(27):17276–84. doi: 10.1039/d2ra01161a (PMC9186304; doi:10.1039/d2ra01161a)
Supplement: RA-012-D2RA01161A-s001 [file RA-012-D2RA01161A-s001.pdf]

Electronic Supplementary Material (ESI) for RSC Advances.
This journal is © The Royal Society of Chemistry 2022

Supplementary Information

for

Subchronic toxicity of magnesium oxide nanoparticles to *Bombyx mori* silkworm

Lin Ma[*#a], Vivian Andoh[#a,b], Zhongyuan Shen[a], Haiyan Liu[c], Long Li[a], Keping Chen[*b]

[a]*College of Biotechnology, Jiangsu University of Science and Technology, Zhenjiang Jiangsu, 212001, P. R. China*

[b]*Institute of Life Science, Jiangsu University, Zhenjiang Jiangsu, 212013, P. R. China*

[c]*Tea and Food Science and Technology Institute, Jiangsu Vocational College of Agriculture and Forestry, Jurong 212400, China*

Email: Lin Ma*-ma_lin_1988@126.com; Keping Chen-kpchen@ujs.edu.cn

*Corresponding author.

# These authors contributed equally to this work.

1. Experimental section

1.1. The characterization of the MgO NPs

Ultraviolet-visible (UV-vis) absorption spectrometry (Ultrospec7000, Biochrom, UK) was used to obtain the absorbance spectrum of the MgO NPs. A MIRA3 SEM (scanning electron microscope, Tescan, Czech Republic) was used to study the size and shape of the MgO NPs. High-resolution transmission electron microscope (TEM)

was further used to characterize the size, shape and lattice fringe of the MgO NPs, using a FEI Talos F200X electron microscope (FEI, America) with a 200 kV acceleration voltage. TEM-EDS (Energy dispersive X-ray spectroscopy) point analysis was also used for element identification in the NP.

1.2. Tensile test

The mechanical parameters of the degummed silks were collected by the college of textile and clothing engineering of Soochow University, *via* an Instron 3365 universal testing machine (Instron, America), the gauge length was 500 mm, extension rate was 500 mm min^{-1}, 20 specimens were measured per group, the whole experiment was carried out in a constant temperature and humidity lab (20 °C, 65% RH).

1.3. Silkworm rearing

Silkworms (*Bombyx mor*i, jingsong × haoyue) were hatched in a climatic chamber under 25 °C and 70-80% RH, newly hatched silkworm larvae were fed with finely chopped fresh mulberry leaves until the 3rd instar, under 27 °C and 80-90% RH. 4th and 5th instar silkworm larvae were transferred into bamboo trays and fed directly with fresh mulberry leaves, under 26 °C and 70-75% RH.

1.4. Silk reeling

Cocoons were reeled to remove the sericin coated on the surface of silk fibroin, through a XJ401 automatic cocoon reeling apparatus (Hangzhou Feiyu technological engineering Co., Ltd., China). In brief, cocoons were dried in a drying oven for 4 h at 80 °C, which was softened in boiling water for a few minutes and transferred into hot water (70 °C). The treated cocoons were finally reeled by an automatic cocoon reeling

apparatus, then shade dried. The cocoons were reeled by using 5 together at a time, which means that the finally obtained degummed silk fiber is a bunch of 5 single silk fibers.

2. Results and Discussion

2.1. The characterization of the MgO NPs

UV-vis absorption spectrometry was employed to characterize the optical response of the MgO NPs, the result displayed in **Fig. S1** shows that the maximum absorption peak is located around 260 nm, corresponding to the dipole resonance of MgO NPs, usually ranged between 260 to 280 nm [**1, 2**]. SEM (scanning electron microscope) and high-resolution TEM (transmission electron microscope) were used to study the geometrical and morphological characteristics of the MgO NPs, and the results can be seen in **Fig. S2a-c**. The SEM result in **Fig. S2a** shows that the MgO NPs are three-dimensional nanomaterials with regular structure. The TEM results in **Fig. S2b, c** show that the shape of MgO NPs is random while some are hexagonal. Calculated by Nano-measure software (Version 1.2), it is estimated that the size of MgO NPs is ranged from 11 to 49 nm, with an approximate average size of 23 nm (the insert in **Fig. 2Sb**). The lattice fringe of the MgO NP with 0.21nm distance, which is assigned to the (200) plane, is clearly seen in **Fig. S2c**, proving its good crystallization. TEM-EDS (Energy dispersive X-ray spectroscopy) point analysis was used to characterize the element composition in the MgO NPs (**Fig. 2Sd**). The result shows that Mg and O are the main contents in the NP, with an atomic fraction of 40.07% (Mg) and 59.93% (O), respectively. Overall, the results prove that the NPs used in this assay is MgO

NPs, with an average size around 20 nm.

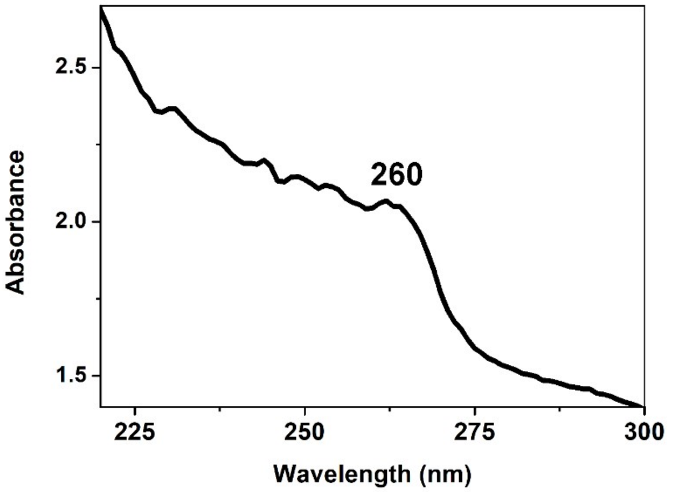

Fig. S1 UV-vis absorbance spectrum of MgO NPs.

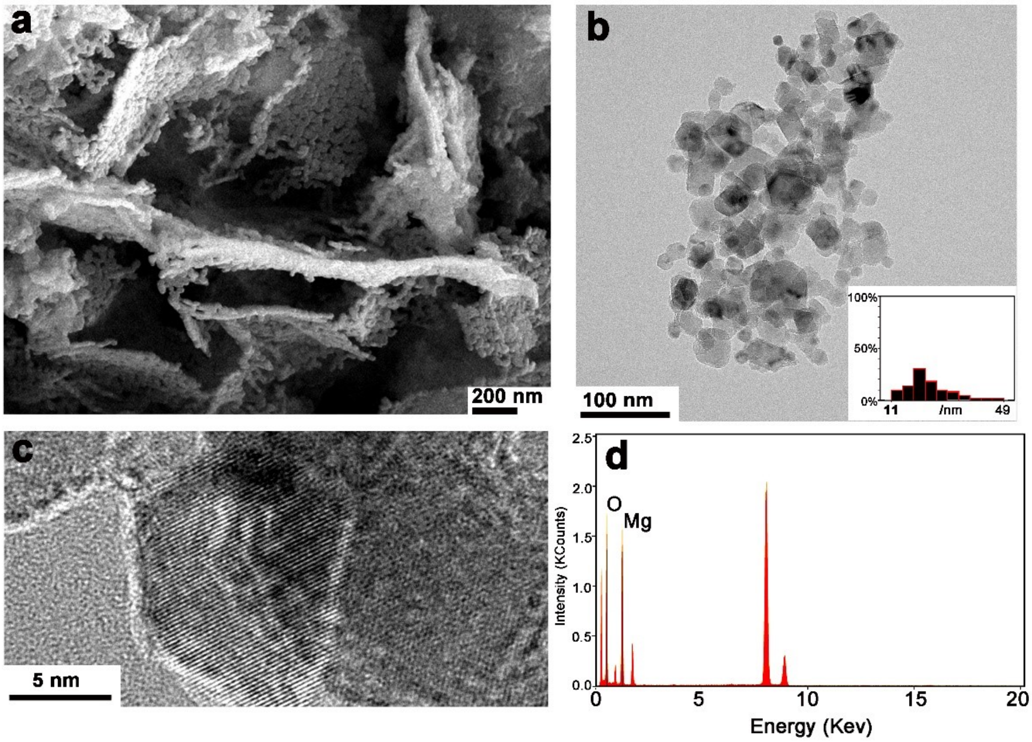

Fig. S2 SEM (a), TEM(b) and high resolution-TEM(c) images of MgO NPs. (d) EDS spectra for MgO NPs.

2.2. The comparison of the average cocoon weight and silk diameter from different groups

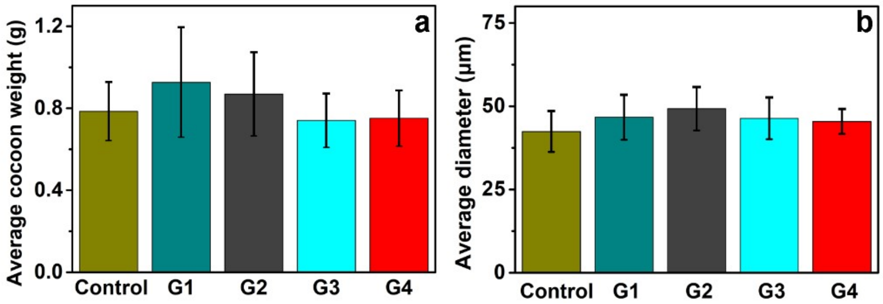

Fig. S3 The comparison of the average cocoon weight (ACW) and average silk diameter (ASD) from different groups. The data of ACW are calculated based on 20 samples, the one of ASD are calculated by the measurement of 10 single silk fibers. The error bars represent the standard deviation of the cocoon weight (a) and silk diameter (b), respectively.

2.3. Mechanical properties of different silks

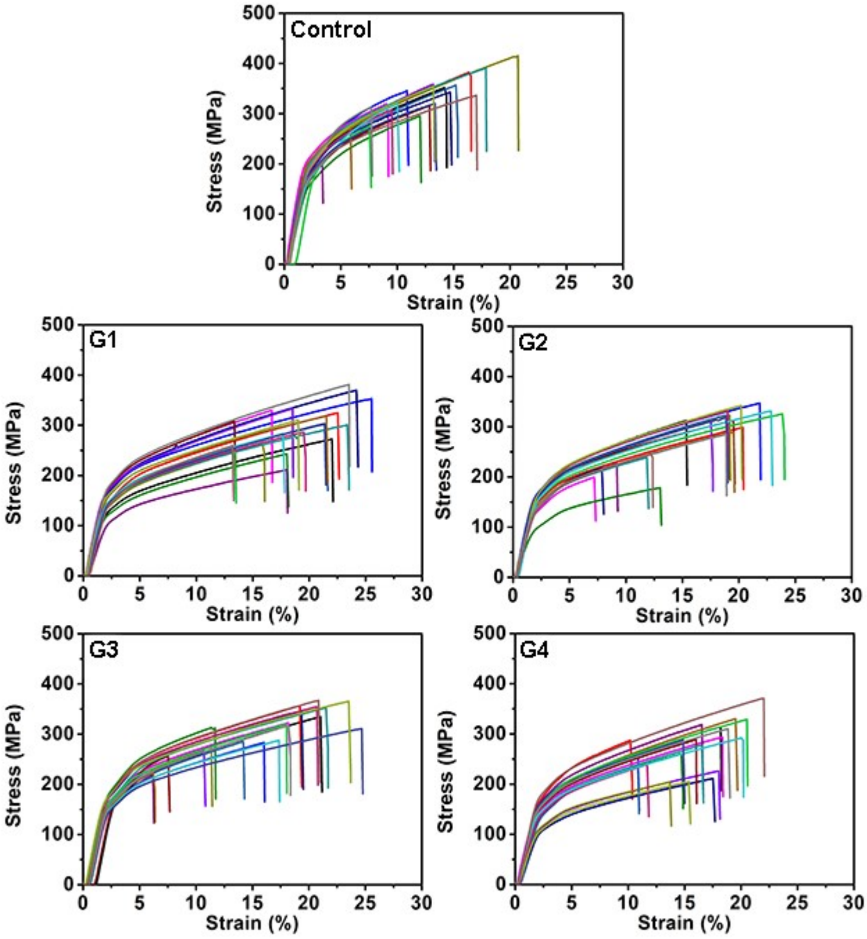

Fig. S4 Strain-Stress curves of different silk samples

Table S1. Mechanical properties of silks

Sample	Elongation at break (%)	Breaking strength (MPa)	Toughness modulus (MPa)
Control	12.23	329.01	30.74
G1	19.43	300.15	44.03
G2	16.87	285.93	37.88
G3	16.32	307.67	38.91
G4	16.22	276.95	33.82

2.4. Global gene expression changes with RNA-SEQ

2.4.1. The expression changes of genes related to metabolism, immune and xenobiotics biodegradation

Table S2. The expression changes of genes related to metabolism

ID	Fold change (G1/control)	P Value	FDR	Description
ncbi_101744519	3.70	1.97×10^{-10}	5.09×10^{-8}	neutral ceramidase
ncbi_101742351	6.14	9.57×10^{-5}	0.00298	heparan-alpha-glucosaminide N-acetyltransferase
ncbi_101737000	3.33	6.86×10^{-8}	9.53×10^{-6}	pancreatic triacylglycerol lipase-like

ncbi_101744377	100	5.87×10^{-6}	0.000363	neutral ceramidase
ncbi_101738716	2.49	3.29×10^{-7}	3.68×10^{-5}	putative fatty acyl-CoA reductase CG5065
ncbi_101743171	160	6.31×10^{-5}	0.00215	acyl-CoA Delta (11) desaturase-like
ncbi_101739150	3.30	0.000280	0.00646	lipase member I-like
ncbi_101736399	2.19	0.00117	0.0183	solute carrier family 22 member 5
ncbi_101745064	2.89	0.00159	0.0221	putative phosphatidate phosphatase
MSTRG.18733	623	5.87×10^{-06}	0.000363	cytochrome P450, partial [*Bombyx mori*]
ncbi_101745761	2.24	0.000390	0.00834	dehydrogenase/reductase SDR family member 4
ncbi_101746769	2.19	0.000214	0.00526	N-acetylglucosaminyl-phosphatidylinositol de-N-acetylase
ncbi_101744960	2.28	7.66×10^{-7}	7.33×10^{-5}	alanine--glyoxylate aminotransferase 2, mitochondrial
ncbi_101744138	4.55	0.00222	0.0278	probable alpha-glucosidase Os06g0675700
ncbi_101741195	3.85	1.24×10^{-6}	0.000112	probable beta-hexosaminidase fdl
ncbi_100101196	2.11	0.00278	0.0321	beta-N-acetylglucosaminidase 2 precursor
ncbi_110385268	3.21	0.00478	0.0469	potassium voltage-gated channel protein Shal-like

ncbi_732849	2.34	0.000587	0.0112	fibroblast growth factor receptor precursor
ncbi_101744393	7.93	0.000180	0.00470	LOW QUALITY PROTEIN: chitooligosaccharidolytic beta-N-acetylglucosaminidase-like
ncbi_101739565	110	0.00138	0.0199	aldose reductase
ncbi_101744767	83.3	0.00259	0.0303	alanine--glyoxylate aminotransferase 2, mitochondrial-like
ncbi_101736038	23.3	0.00259	0.0303	para-nitrobenzyl esterase
ncbi_100329149	2.26	1.47×10^{-6}	0.000129	aliphatic nitrilase
ncbi_101736396	4.50	0.00106	0.0171	alpha-amylase 4N
ncbi_101743752	53.3	0.00259	0.0303	elongation of very long chain fatty acids protein 7
ncbi_101735436	76.7	0.00490	0.0469	glutamine synthetase 2 cytoplasmic
ncbi_101740654	53.3	0.000395	0.00834	fatty acyl-CoA reductase wat
ncbi_101742671	0.492	0.000196	0.00506	probable allantoicase
MSTRG.17018	0.313	3.41×10^{-6}	0.000249	aminopeptidase N precursor [*Bombyx mori*]
ncbi_101745114	0.401	3.95×10^{-5}	0.00158	decaprenyl-diphosphate synthase subunit 2
ncbi_101736031	0.133	5.81×10^{-5}	0.00208	LOW QUALITY PROTEIN:

				alpha-N-acetylgalactosaminidase
ncbi_101739517	0.183	0.000114	0.00337	N-acetylneuraminate lyase
ncbi_101736026	0.365	0.000222	0.00538	adenine phosphoribosyltransferase
ncbi_101735471	0.210	0.000130	0.00369	methylenetetrahydrofolate reductase
ncbi_101742863	0.471	0.00236	0.0291	fatty acyl-CoA reductase wat-like
ncbi_101743809	0.264	3.88×10^{-6}	0.000277	vanin-like protein 1
ncbi_100862823	0.0120	0.00138	0.0199	UDP-glycosyltransferase UGT47A1 precursor
ncbi_692403	0.477	0.000441	0.00901	chitinase-related protein 1 precursor
ncbi_101743711	0.490	0.000305	0.00683	lipase 1
ncbi_101744242	0.333	1.69×10^{-5}	0.000818	probable peroxisomal acyl-coenzyme A oxidase 1
ncbi_105841519	0.342	0.00150	0.0211	lipase 1-like
ncbi_105842081	0.457	0.00338	0.0370	adenylate cyclase type 3
ncbi_105841520	0.394	0.00513	0.0480	lipase 1
ncbi_692793	0.261	0.000421	0.00871	arginase
ncbi_101740270	0.0500	0.00490	0.0469	uncharacterized protein LOC101740270
ncbi_101747146	0.0115	0.00490	0.0469	uncharacterized protein LOC101747146
ncbi_101744260	0.125	0.00379	0.0400	putative fatty acyl-CoA reductase

				CG5065
ncbi_101738818	0.324	0.00309	0.0346	NADH dehydrogenase [ubiquinone] 1 alpha subcomplex subunit 6-like
ncbi_101743673	0.388	0.000634	0.0119	catalase
ncbi_110384899	0.0125	0.00490	0.0469	dehydrogenase/reductase SDR family member 13-like
ncbi_101745539	0.465	0.00122	0.0189	thiamin pyrophosphokinase 1
ncbi_110385499	0.466	5.82×10^{-6}	0.000363	LOW QUALITY PROTEIN: uncharacterized protein LOC110385499
ncbi_101742639	0.0300	0.00490	0.0469	high-affinity choline transporter 1
ncbi_101742625	0.333	0.00536	0.0494	fatty acid synthase
ncbi_101741329	0.267	5.32×10^{-10}	1.22×10^{-7}	LOW QUALITY PROTEIN: catalase
ncbi_100862808	0.493	2.57×10^{-5}	0.00113	UDP-glycosyltransferase UGT33R1 precursor
ncbi_101739999	0.475	0.00350	0.0379	putative fatty acyl-CoA reductase CG5065
ncbi_100862782	0.454	3.01×10^{-6}	0.000230	c-myc
ncbi_100141446	0.216	3.84×10^{-10}	9.33×10^{-8}	glutathione S-transferase epsilon 3
ncbi_100329150	0.426	3.99×10^{-7}	4.40×10^{-5}	carboxyl/cholinesterase 1 precursor

ncbi_101739608	0.499	3.41×10^{-5}	0.00143	1-acyl-sn-glycerol-3-phosphate acyltransferase alpha-like
ncbi_101735916	0.391	5.20×10^{-7}	5.48×10^{-5}	fatty acyl-CoA reductase wat
ncbi_101737703	0.392	6.38×10^{-7}	6.40×10^{-5}	glycine cleavage system H protein, mitochondrial
ncbi_101738534	0.248	8.16×10^{-10}	1.84×10^{-7}	acyl-CoA Delta(11) desaturase-like
ncbi_101738415	0.496	4.98×10^{-5}	0.00187	dehydrodolichyl diphosphate synthase complex subunit Nus1
ncbi_101745831	0.486	2.78×10^{-5}	0.00121	putative fatty acyl-CoA reductase CG5065
ncbi_101737440	0.338	1.62×10^{-10}	4.34×10^{-8}	esterase FE4-like
ncbi_101738177	0.443	2.94×10^{-5}	0.00128	triokinase/FMN cyclase
ncbi_100862842	0.448	3.38×10^{-6}	0.000248	UDP-glycosyltransferase UGT33R2 precursor
ncbi_101744378	0.426	2.80×10^{-5}	0.00122	putative fatty acyl-CoA reductase CG5065
ncbi_101736595	0.0115	1.05×10^{-5}	0.000579	uncharacterized protein LOC101736595
ncbi_101736222	0.473	8.00×10^{-6}	0.000468	molybdopterin synthase catalytic subunit
ncbi_101745478	0.485	1.30×10^{-5}	0.000685	GTP 3',8-cyclase, mitochondrial

ncbi_101746910	0.484	1.13×10^{-5}	0.000611	glucose dehydrogenase [FAD, quinone]
ncbi_100642185	0.194	0.000273	0.00635	soluble guanylyl cyclae alpha-1 subunit
ncbi_105842833	0.00155	0.000395	0.00834	phosphopantothenate--cysteine ligase-like
MSTRG.7665	0.307	0.000368	0.00794	mariner transposase [*Bombyx mori*]
ncbi_105842680	0.0120	0.000116	0.00337	uncharacterized protein LOC105842680
ncbi_101736644	0.188	9.18×10^{-8}	1.21×10^{-5}	lysosomal alpha-mannosidase
ncbi_105842722	0.393	0.000167	0.00447	GPI-anchored wall transfer protein 1-like
ncbi_101737058	0.136	1.73×10^{-6}	0.000148	carbohydrate sulfotransferase 11
ncbi_101735627	0.000952	4.63×10^{-24}	5.31×10^{-21}	beta-1,4-galactosyltransferase 7
ncbi_692978	0.442	6.75×10^{-5}	0.00224	thymidylate synthase
ncbi_101740404	0.408	4.58×10^{-5}	0.00175	methionyl-tRNA formyltransferase, mitochondrial
ncbi_101743443	0.335	1.48×10^{-5}	0.000750	mucin-2
ncbi_692458	0.409	4.03×10^{-5}	0.00159	uncharacterized protein LOC692458
ncbi_101744495	0.00577	6.31×10^{-5}	0.00215	N-acetylneuraminate lyase
ncbi_693000	0.0656	8.87×10^{-6}	0.000508	yellow-fa precursor

ncbi_101744555	0.484	0.000207	0.00526	3-hydroxyacyl-CoA dehydrogenase type-2
ncbi_101746170	0.100	1.86×10^{-11}	5.73×10^{-9}	catalase
ncbi_101741806	0.488	0.000203	0.00521	protein 5NUC
MSTRG.9400	0.0115	0.000395	0.00834	PREDICTED: organic cation transporter protein-like [Amyelois transitella]
ncbi_101738709	0.271	5.18×10^{-7}	5.48×10^{-5}	carbonyl reductase [NADPH] 3
ncbi_100500758	0.426	0.000284	0.00651	UDP-glucosyltransferase precursor
ncbi_101743127	0.496	0.000695	0.0128	DNA primase large subunit

Table S3. The expression changes of genes related to immune

ID	Fold change (G1/control)	P Value	FDR	Description
ncbi_692420	0.450	1.46×10^{-6}	0.000129	heat shock protein 83
ncbi_100862772	0.0563	1.36×10^{-47}	8.60×10^{-44}	heat shock protein 68
ncbi_101738177	0.443	2.94×10^{-5}	0.00128	triokinase/FMN cyclase
ncbi_101736595	0.0115	1.06×10^{-5}	0.000579	uncharacterized protein LOC101736595
ncbi_101745500	0.279	8.75×10^{-10}	1.94×10^{-7}	heat shock 70 kDa protein
ncbi_100147699	0.330	2.30×10-8	3.93×10^{-6}	chymotrypsin inhibitor SCI-III precursor
MSTRG.17018	0.313	3.41×10^{-6}	0.000249	aminopeptidase N precursor [*Bombyx mori*]

ncbi_100642185	0.194	0.000273	0.00635	soluble guanylyl cyclae alpha-1 subunit
ncbi_693028	0.304	0.00166	0.0228	cecropin-B precursor
ncbi_733093	0.284	2.38×10^{-13}	9.72×10^{-11}	heat shock protein 70 A1
ncbi_101739681	0.427	0.00171	0.0231	cecropin-B
ncbi_105842081	0.457	0.00338	0.0370	adenylate cyclase type 3
MSTRG.9759	0.365	0.00272	0.0315	serine protease inhibitor 13 precursor [*Bombyx mori*]
ncbi_778518	0.403	5.04×10^{-7}	5.48×10^{-5}	myosin regulatory light chain 2
ncbi_101735832	0.422	2.59×10^{-7}	3.00×10^{-5}	heat shock protein 70 A2
ncbi_692822	0.433	6.85×10^{-7}	6.71×10^{-5}	heat shock protein 70
MSTRG.18492	2.52	1.26×10^{-7}	$1.57\times10-^{5}$	hemocytin [*Bombyx mori*]
ncbi_692561	2.67	5.21×10^{-5}	0.00192	notch homolog
ncbi_100188966	2.61	0.00119	0.0185	scavenger receptor type C precursor
ncbi_101737265	40.0	0.000213	0.00524	integrin alpha-PS4-like
ncbi_101740436	233	0.00138	0.0199	signal transducer and transcription activator-like
ncbi_101740789	2.39	0.00354	0.0382	phosrestin-2
ncbi_101745064	2.89	0.00159	0.0221	putative phosphatidate phosphatase

Table S4. The expression changes of genes related to xenobiotics biodegradation

ID	Fold change	P Value	FDR	Description

	(G1/control)			
ncbi_100141446	0.216	3.84×10^{-10}	9.33×10^{-8}	glutathione S-transferase epsilon 3
ncbi_100329150	0.426	3.99×10^{-7}	4.40×10^{-5}	carboxyl/cholinesterase 1 precursor
ncbi_101744852	0.492	4.61×10^{-5}	0.00176	cytochrome P450 4V2
ncbi_100862842	0.449	3.38×10^{-6}	0.000248	UDP-glycosyltransferase UGT33R2 precursor
ncbi_780858	0.427	2.53×10^{-6}	0.000205	cytochrome P450 6AB4
ncbi_100127103	0.0732	6.56×10^{-5}	0.00220	cytochrome P450 Cyp305B1 precursor
ncbi_101738709	0.271	5.18×10^{-7}	5.48×10^{-5}	carbonyl reductase [NADPH] 3
ncbi_100500758	0.426	0.000284	0.00651	UDP-glucosyltransferase precursor
ncbi_101737893	0.338	9.32×10^{-5}	0.00292	cytochrome P450 6B2-like
ncbi_101739162	0.364	1.55×10^{-5}	0.000774	probable cytochrome P450 9f2
ncbi_100862823	0.0120	0.00138	0.0199	UDP-glycosyltransferase UGT47A1 precursor
ncbi_101744935	0.129	0.00137	0.0199	cytochrome P450 4V2
ncbi_100862808	0.493	2.57×10^{-5}	0.00113	UDP-glycosyltransferase

				UGT33R1 precursor
ncbi_101742830	271	5.94×10^{-60}	7.50×10^{-56}	cytochrome P450 9e2
MSTRG.18733	623	5.87×10^{-6}	0.000363	cytochrome P450, partial [*Bombyx mori*]
ncbi_101736038	23.3	0.00259	0.0303	para-nitrobenzyl esterase
ncbi_100329149	2.26	1.47×10^{-6}	0.000129	aliphatic nitrilase
ncbi_105842283	187	0.000116	0.00337	probable cytochrome P450 49a1

2.4.2. GO and KEGG analysis of global gene expression changes

Table S5. GO enrichment (TOP 20 most significantly changed ones)

GO ID	GO term (level 1)	GO term (level 2)	P value	P. adjust	Numbers up	Numbers down
GO:0044765	biological Process	single-organism transport	0.06	0.4176	0	7
GO:0006820	biological Process	anion transport	0.06	0.4176	0	1
GO:0006839	biological Process	mitochondrial transport	0.06	0.4176	0	1
GO:0007268	biological Process	synaptic transmission	0.06	0.4176	0	1
GO:0007271	biological Process	synaptic transmission, cholinergic	0.06	0.4176	0	1
GO:0008033	biological Process	tRNA processing	0.06	0.4176	0	1
GO:0009157	biological Process	deoxyribonucleoside monophosphate biosynthetic process	0.06	0.4176	0	1
GO:0009162	biological Process	deoxyribonucleoside monophosphate metabolic process	0.06	0.4176	0	1
GO:0009176	biological Process	pyrimidine deoxyribonucleoside monophosphate metabolic process	0.06	0.4176	0	1
GO:0009177	biological Process	pyrimidine deoxyribonucleoside monophosphate biosynthetic process	0.06	0.4176	0	1

GO:0010817	biological Process	regulation of hormone levels	0.06	0.4176	0	1
GO:0015711	biological Process	organic anion transport	0.06	0.4176	0	1
GO:0015849	biological Process	organic acid transport	0.06	0.4176	0	1
GO:0019098	biological Process	reproductive behavior	0.06	0.4176	1	0
GO:0032222	biological Process	regulation of synaptic transmission, cholinergic	0.06	0.4176	0	1
GO:0033057	biological Process	multicellular organism reproductive behavior	0.06	0.4176	1	0
GO:0034470	biological Process	ncRNA processing	0.06	0.4176	0	1
GO:0042445	biological Process	hormone metabolic process	0.06	0.4176	0	1
GO:0042446	biological Process	hormone biosynthetic process	0.06	0.4176	0	1
GO:0044705	biological Process	multi-organism reproductive behavior	0.06	0.4176	1	0
GO:0015291	molecular Function	secondary active transmembrane transporter activity	0.008	0.305357	0	3
GO:0015929	molecular Function	hexosaminidase activity	0.029	0.305357	2	0
GO:0022804	molecular Function	active transmembrane transporter activity	0.034	0.305357	0	3

GO:0005215	molecular Function	transporter activity	0.049	0.305357	0	5
GO:0001871	molecular Function	pattern binding	0.057	0.305357	1	0
GO:0003954	molecular Function	NADH dehydrogenase activity	0.057	0.305357	0	1
GO:0005310	molecular Function	dicarboxylic acid transmembrane transporter activity	0.057	0.305357	0	1
GO:0005342	molecular Function	organic acid transmembrane transporter activity	0.057	0.305357	0	1
GO:0008509	molecular Function	anion transmembrane transporter activity	0.057	0.305357	0	1
GO:0008514	molecular Function	organic anion transmembrane transporter activity	0.057	0.305357	0	1
GO:0016655	molecular Function	oxidoreductase activity, acting on NAD(P)H, quinone or similar compound as acceptor	0.057	0.305357	0	1
GO:0016782	molecular Function	transferase activity, transferring sulfur-containing groups	0.057	0.305357	0	1
GO:0046943	molecular Function	carboxylic acid transmembrane transporter activity	0.057	0.305357	0	1
GO:0050136	molecular Function	NADH dehydrogenase (quinone) activity	0.057	0.305357	0	1
GO:0008395	molecular Function	steroid hydroxylase activity	0.112	0.502941	1	0

GO:0009975	molecular Function	cyclase activity	0.112	0.502941	0	1
GO:0022857	molecular Function	transmembrane transporter activity	0.114	0.502941	0	4
GO:0004553	molecular Function	hydrolase activity, hydrolyzing O-glycosyl compounds	0.127	0.529167	2	0
GO:0005048	molecular Function	signal sequence binding	0.163	0.547826	0	1
GO:0016651	molecular Function	oxidoreductase activity, acting on NAD(P)H	0.163	0.547826	0	1
GO:0031224	cellular Component	intrinsic component of membrane	0.0054	0.1173	0	10
GO:0044425	cellular Component	membrane part	0.0102	0.1173	0	10
GO:0016020	cellular Component	membrane	0.0659	0.505233	0	10
GO:0097458	cellular Component	neuron part	0.1064	0.596083	0	1
GO:0015630	cellular Component	microtubule cytoskeleton	0.1541	0.596083	2	0
GO:0048500	cellular Component	signal recognition particle	0.1555	0.596083	0	1
GO:0005856	cellular Component	cytoskeleton	0.3207	1	2	0
GO:0005737	cellular Component	cytoplasm	0.4828	1	0	2

GO:0043228	cellular Component	non-membrane-bounded organelle	0.6942	1	2	0
GO:0043232	cellular Component	intracellular non-membrane-bounded organelle	0.6942	1	2	0
GO:0044444	cellular Component	cytoplasmic part	0.7931	1	0	1
GO:0030529	cellular Component	intracellular ribonucleoprotein complex	0.8331	1	0	2
GO:1990904	cellular Component	ribonucleoprotein complex	0.8331	1	0	2
GO:0043229	cellular Component	intracellular organelle	0.8631	1	4	2
GO:0043226	cellular Component	organelle	0.8686	1	4	2
GO:0043231	cellular Component	intracellular membrane-bounded organelle	0.9049	1	2	2
GO:0005622	cellular Component	intracellular	0.9054	1	4	6
GO:0005623	cellular Component	cell	0.9184	1	4	7
GO:0044464	cellular Component	cell part	0.9184	1	4	7
GO:0032991	cellular Component	macromolecular complex	0.9213	1	2	2

94 **Table S6.** KEGG pathway analysis of differently expressed genes

KEGG_A_class	KEGG_B_class	Pathway	Control-vs-G1(199)	All (4233)	P value	Q value
Metabolism	Lipid metabolism	Cutin, suberine and wax biosynthesis	8	34	0.000131	0.0310
Organismal Systems	Aging	Longevity regulating pathway-worm	15	115	0.000265	0.0310
Human Diseases	Neurodegenerative diseases	Prion diseases	7	30	0.000369	0.0310
Organismal Systems	Immune system	Antigen processing and presentation	6	23	0.000516	0.0325
Human Diseases	Cancers	Transcriptional misregulation in cancers	13	105	0.00112	0.0567
Organismal Systems	Aging	Longevity regulating pathway-multiple species	12	103	0.00293	0.123
Cellular Processes	Cellular community-eukaryotes	Gap junction	8	59	0.00582	0.206

Cellular Processes	Transport and catabolism	Peroxisome	13	129	0.00702	0.206
Human Diseases	Infectious diseases	Legionellosis	6	38	0.00784	0.206
Human Diseases	Infectious diseases	Measles	7	50	0.00816	0.206
Metabolism	Glycan biosynthesis and metabolism	Glycosphingolipid biosynthesis-globo and isoglobo series	3	10	0.00961	0.220
Human Diseases	Infectious diseases	Pathogenic Escherichia coli infection	6	41	0.0114	0.238
Environmental Information Processing	Signal transduction	MAPK signaling pathway	12	135	0.0238	0.426
Metabolism	Lipid metabolism	Steroid biosynthesis	4	24	0.0240	0.426
Metabolism	Metabolism of cofactors and vitamins	One carbon pool by folate	3	14	0.0254	0.426

Organismal Systems	Digestive system	Fat digestion and absorption	6	51	0.0309	0.431
Human Diseases	Infectious diseases	Toxoplasmosis	6	51	0.0309	0.431
Metabolism	Lipid metabolism	Glycerolipid metabolism	7	65	0.0315	0.431
Organismal Systems	Endocrine system	Estrogen signaling pathway	7	67	0.0364	0.431
Metabolism	Metabolism of cofactors and vitamins	Pantothenate and CoA biosynthesis	3	16	0.0364	0.431
Genetic Information Processing	Folding, sorting and degradation	Sulfur relay system	2	7	0.0395	0.431
Metabolism	Carbohydrate metabolism	Amino sugar and nucleotide sugar metabolism	6	54	0.0395	0.431
Metabolism	Glycan biosynthesis and metabolism	Other glycan degradation	4	28	0.0400	0.431

Metabolism	Glycan biosynthesis and metabolism	Glycosaminoglycan degradation	3	17	0.0428	0.431
Organismal Systems	Immune system	Complement and coagulation cascades	3	17	0.0428	0.431
Metabolism	Carbohydrate metabolism	Glyoxylate and dicarboxylate metabolism	5	42	0.0452	0.438

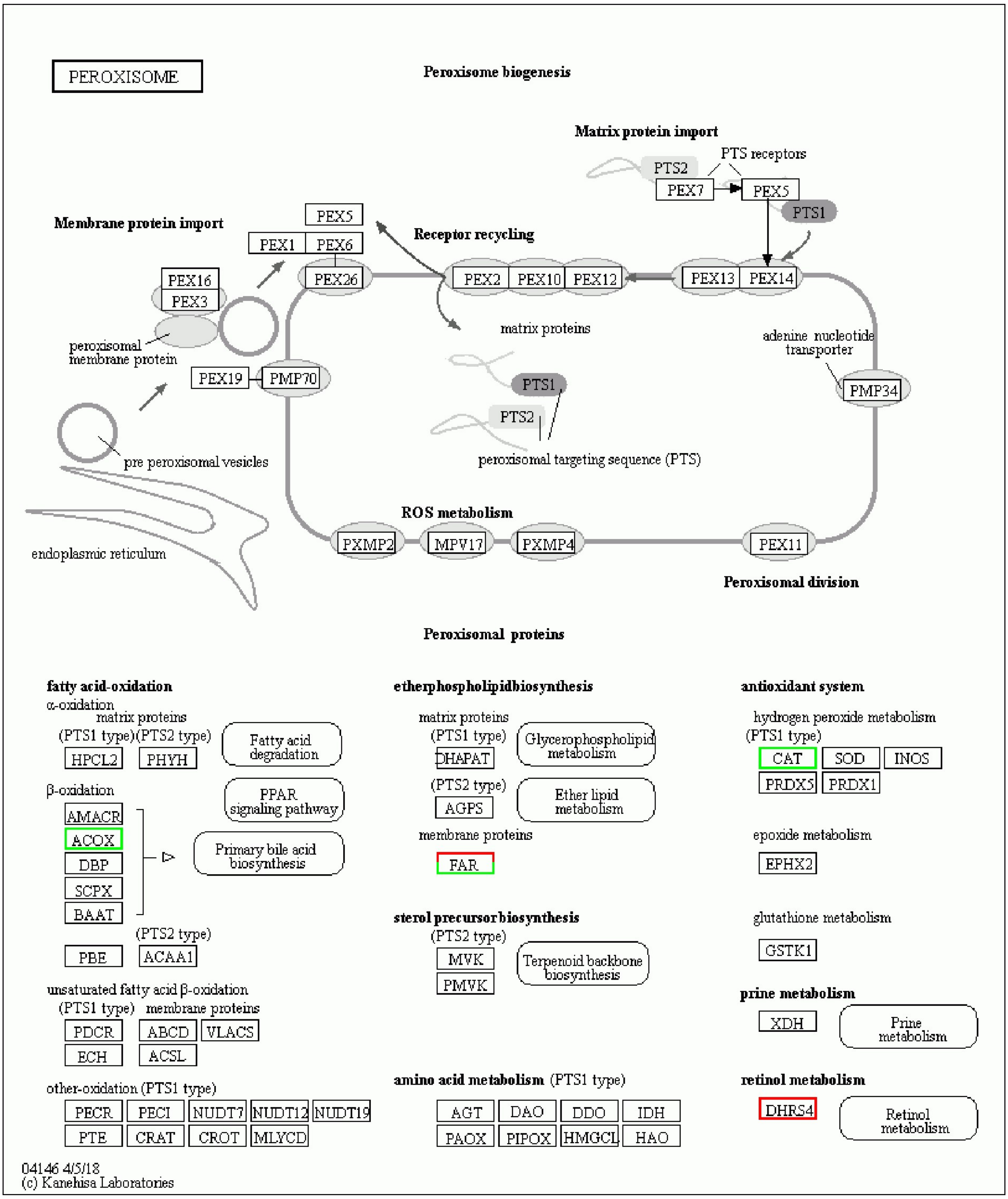

Fig. S5 KEGG pathways of the peroxisome in MgO NPs group *vs* control group. Red color means up-regulated, green color means down-regulated.

Reference

[1] E. R. Essien, V. N. Atasie, A. O. Okeafor, D. O. Nwude. Biogenic synthesis of magnesium oxide nanoparticles using *Manihot esculenta* (Crantz) leaf extract. *Int.*

Nano Lett., 2020, 10, 43–48.

[2] M. Vergheese, S. Kiran-Vishal. Green synthesis of magnesium oxide nanoparticles using *Trigonella foenum-graecum* leaf extract and its antibacterial activity. *J. Pharmacogn. Phytochem.* 2018, 7, 1193–1200.
